# Pulmonary Tuberculosis

**Published:** 1894-01-13

**Authors:** 


### Medical Progress.

### PULMONARY TUBERCULOSIS.

### General Therapeutics.

Much has been done by Dr. J. K. Fowler in forming current ideas as to the clinical course of tubercular disease of the lungs, and it is largely due to his monograph on "Arrested Pulmonary Tuberculosis" that we have come to look upon true tubercular phthisis as a curable disease. His further contribution on "The Varieties of Pulmonary Tuberculosis" directs attention to the indefinite classification of varieties of phthisis hitherto in vogue.

"Now it is certainly true of the great majority of cases that the clinical course is so fluctuating in character, and the pathological lesions are so variously combined, as to render any closer definition than is conveyed by the term 'chronic pulmonary tuberculosis' unnecessary. There remain, however, a certain number to which separate names may, I believe, with advantage be given. For these I have proposed the following: (1) Miliary tuberculosis of the lungs; (2) caseous tuberculosis of the lungs; (3) fibroid tuberculosis of the lungs. The pathological features of tuberculosis lesions, upon which this classification is based, depend upon, and are at the same time a measure of, the activity of the morbid process; they may, therefore, be used as clinical terms to express the varying rates of progress of the disease."

Aero therapeutics in lung disease formed the subject of Dr. Williams' Lumleian lectures. He deals with the question of barometric pressure in its relation to health in disease, and describing the various methods by which the effects of compressed air may be utilised clinically, and summarises his results of the effects of compressed air in lung diseases. His conclusions in the present instance are based on cases from his private notebooks, and on sixty-six patients at the Brompton Hospital.

*Asthma.*—Of bronchial asthma there were fifteen patients—ten males and five females. In seven asthma was largely complicated with emphysema. The average number of baths taken was twelve to fifteen. Of these patients twelve improved, and three did not improve. Out of eleven whose weights were taken, nine gained weight—on an average four and two-thirds pounds—and two lost. The measurement of the circumference of the thorax at various levels was made in seven patients, before and after the baths, and the circumference had increased in four and diminished in three. The spirometer showed an increase of 25 to 33 per cent. in the three cases in which this test was applied. The principal effect of the baths on asthma appears to be sedative to the pulmonary plexuses of nerves and the pneumogastric. The attacks are rendered less severe, and, after a course of twenty or thirty baths, the intervals between the attacks become much longer. He does not remember one case where a complete cure was effected, but recollects several where the patient remained free for months, and, in one instance, for years, from asthma. The effect on the paroxysm is immediate and wonderfully efficacious, and many asthmatics have expressed the wish that they could live in the bath and thus be freed from their sufferings. The effect on the emphysema accompanying it is to reduce it, as percussion and auscultation show.

*Chronic Bronchitis and Emphysema.*—In chronic bronchitis and emphysema the effect is satisfactory, the cough diminishes and the expectoration is lessened, weight is gained, breathing is easier, but the great feature is the reduction of the emphysema. Examination of the chest shows diminution of the hyper-resonance and a return of the various displaced organs to their normal positions and cyrtometric measurements give a reduction of the chest's circumference at different levels, a reduction varying from half inch to one and a-half inch. This indicates that a great deal of emphysema, even in adults, is of a temporary nature, produced often by severe paroxysms of coughing or dyspnœa, and capable of reduction if respiration be rendered more easy as in a compressed air atmosphere. In his thirty-three cases of chronic bronchitis and emphysema, who had on an average eighteen baths, fifteen were measured cyrtometrically, and of these eleven were found to have decreased in circumference, and four to have increased; twenty-six of the patients improved generally, five did not improve, and two died—one of heart failure from cardiac dilatation after five baths, and the other from an attack of capillary bronchitis, after having greatly improved from nine baths, when he contracted fresh bronchitis and died later.

*Phthisis.*—Dr. Williams' experience of compressed air in phthisis is not altogether favourable. In nine of the cases he submitted to the bath there was a gain of weight and some diminution of cough and expectoration, and apparently the respiration became freer in the unaffected portions of the lungs, but in two cases the bath appeared to bring on hæmoptysis, and in four patients hæmoptysis came on during the treatment, though it could not be distinctly connected with it. Beyond the opening up or aeration of portions of the lung which had not been brought into play for some time, he could see none of the improvement resulting from compressed air which is so loudly proclaimed by Oertel and Simonoff, neither could he discover that it facilitated the absorption of lung consolidations or infiltration, though cases of this class were submitted to the bath; or, lastly, that it promoted, as Oertel states, the absorption of serious exudation in acute pleurisy, and tended to expand the compressed lung. His experience is that it exercises no effect in expanding a lung which has been compressed with fluid when the fluid has been removed, and, even after a course of air baths steadily persevered in, the fluid will reaccumulate, and will make itself known by indubitable physical signs, and by the diminishing amount of expiratory power as evidenced by the spirometer.

Passing on to the question of climate and high altitude in the treatment of lung disease, Dr. Williams states that, compared with Alpine sanitaria, Colorado offers the advantage of no snow-melting season, and of allowing continuous residence all the year round in a somewhat warmer and dryer climate, but it has the drawbacks of wind and dust, most troublesome in summer. With regard to results, it has not yet produced as favourable statistics as the Swiss. It presents also a great variety of altitude, so that invalids pass their summers camping out in the parks and leading an open-air life, hunting, shooting, and fishing, while they spend their winters in Denver and Colorado springs, or in the foothill towns. But perhaps a greater attraction still to many is that, for a large proportion of patients, Colorado offers a prospect of profitable occupation, whether professional or commercial, and the Western people kindly welcome strangers into their rising State, well knowing that there is room for all within its large confines.

He has found high altitudes beneficial in other diseases besides phthisis, viz., in—1. Imperfect thoracic and pulmonary development. 2. Chronic pneumonia without bronchiectasis. 3. Chronic pleurisy, where the lung is not expanded after the removal of the effusion. 4. Bronchial asthma without emphysema.

These climates are contra-indicated in—1. Phthisis with double cavities. 2. Fibroid phthisis, and all cases where the pulmonary area at sea level hardly suffices for respiratory purposes. 3. Catarrhal and laryngeal phthisis. 4. Acute phthisis of all kinds, and especially when there is great irritability of the nervous system. 5. Phthisis with pyrexia, and in pyrexial cases generally. 6. Emphysema. 7. Chronic bronchitis and bronchiectasis. 8. Diseases of the heart and great vessels, except functional ones; disease of the liver and the kidneys, including all forms of albuminuria (Andrew Clark). 9. Diseases of the brain and spinal cord, and conditions of hypersensibility of the nervous system. 10. Anæmia. 11. In patients of advanced age or too feeble to take exercise.

Comparing home statistics with foreign, Dr. Williams' results are very striking. In general results the home climates yield the smallest percentage of improvement, and the largest of worse; next comes the Riviera, not much better; then, with a rise of 12 per cent. improved are sea voyages, the percentage of worse being still large; high altitudes win easily in all categories, with its 83 per cent. improved, and only 14½ per cent. of worse.

In local results the arrest cases have been made a separate division, but they are also included under the improvement percentage. We see here that the Riviera comes out worst, except for a large number of arrests. Next we find home climates, then, separated by an interval of 14 per cent. more of improved and 12 per cent. less of worse, are sea voyages. The high altitudes again come out *facile princeps* in all categories with favourable percentages, nearly double those of the Riviera and home climates. Looking calmly at these results, it must be admitted that there is strong evidence in favour of high altitude treatment.

The views of so distinguished an authority on the treatment of lung diseases, especially in reference to climate, will have great weight, and his lectures are full of valuable practical information.

Under the term *A Mechanical Treatment of Phthisis*, Mr. Noble Smith has devised a method of improving the general condition of phthisical patients by mechanically expanding the thorax and permanently enlarging the aerial capacity of the lungs, a plan which Mr. Smith has developed from Dr. Silvester's physiological method of treating consumption. His plan of treatment consists in drawing the shoulders backwards by soft shoulder straps to a pad placed centrally between the scapulæ, and permanently held so. There are many so-called braces largely advertised in the present day for effecting this purpose, and if Mr. Smith's plan consisted in nothing more, it would be open to the grave objections which he raises against these patent braces. They pull the shoulders back, and give a false appearance of an improvement, but they do nothing to relieve the chest from the weight of the arms themselves. The new plan consists in keeping the central pad to the level of the shoulders, and so producing a somewhat upward support by carrying a light steel rod down the back to the level of the seat, there to be maintained by a pelvic belt. With this alone we find that there is a tendency in sitting for the spine to project backwards, and in standing for the abdomen to sink forwards, causing a condition of lordosis. To obviate these postures a pad is placed in the centre of the back to prevent the backward projection in sitting, and the belt is placed in front of the abdomen to limit its projection while the patient is standing. Mr. Noble Smith's practital experience of the results of this treatment is of the most satisfactory kind. Although he has not dealt with patients simply for the treatment of phthisis, the cases which he has treated clearly indicate the benefits which such patients would derive from the method.

In advocating this new plan of treatment, Mr. Smith wishes it to be most thoroughly understood that he is not advocating it in the place of other measures.

(*To be continued.*)

